# Team IHMC at the 2020 Cybathlon: a user-centered approach towards personal mobility exoskeletons

**DOI:** 10.1186/s12984-022-01074-8

**Published:** 2022-09-27

**Authors:** Brandon Peterson, Mark Daniel, Vishnu Subra Mani, Brooke Arnold, Travis Craig, Jeremy Gines, Carlos Gonzalez, William Howell, Brandon Shrewsbury, Matthew Bellman, Peter Neuhaus, Robert Griffin

**Affiliations:** 1 Florida Institute for Human and Machine Cognition, Pensacola, FL USA; 2 MYOLYN, Gainesville, FL USA

**Keywords:** Exoskeletons, Assistive devices, Wearable robotics, Cybathlon, Spinal cord injury

## Abstract

**Background:**

The past few decades have seen rapid advancements in exoskeleton technology, with a considerable shift towards applications involving users with gait pathologies. Commercial devices from ReWalk, Ekso Bionics, and Indego, mainly designed for rehabilitation purposes, have inspired the development of many research platforms aimed at extending capabilities for use as safe and effective personal mobility devices. The 2016 Cybathlon featured an impressive demonstration of exoskeletons designed to enable mobility for individuals with spinal cord injury, however, not a single team completed every task and only two completed the stairs. Major improvements were showcased at the 2020 Cybathlon, with seven of the nine teams completing a similar set of tasks. Team IHMC built upon its silver-medal success from 2016 with an upgraded device, Quix.

**Methods:**

Quix features several notable improvements including an additional powered degree of freedom for hip ab/adduction to laterally shift the device and reduce user effort while walking, custom-tailored cuffs and soft goods based on 3D body scans to optimize user comfort, and a streamlined testing pipeline for online tuning of gait parameters.

**Results:**

Team IHMC finished in fourth place behind the teams from EPFL and Angel Robotics. Although we suffered from a considerably slower flat-ground walking speed, our pilot reported marked improvements in overall effort, comfort, and ease-of-use compared to our previous device.

**Conclusions:**

Clear progress in exoskeleton development has been exhibited since the inaugural Cybathlon, with tasks involving rough terrain, stairs, and ramps now posing little threat to most of the competitors. As a result, the layout of the powered exoskeleton course will likely undergo significant modifications to further push the devices towards suitability for personal everyday use. The current tasks do not address the issue of donning and doffing, nor do they simulate a scenario similar to maneuvering a kitchen to prepare a meal, for example. An additional limitation that may be more difficult to test in a competition setting is the required upper-body effort to manipulate the device in an effective manner.

## Background

Exoskeletons became a reality in 1890 with the development of Nicholas Yagin’s apparatus for facilitating walking, running, and jumping by storing energy in gas accumulators [1]. This idea of augmenting human abilities remained the focus for the design of many exoskeletons over the following century. Only within the past few decades have researchers begun developing exoskeletons as rehabilitation devices to address the lack of mobility in individuals with spinal cord injury (SCI), stroke, and other gait pathologies. An estimated 296,000 people with SCI are living in the United States alone, with roughly 18,000 new cases each year [2]. That number contributes to the 75 million people worldwide in need of wheelchair, of which only 5–15% are fortunate enough to gain access [3]. While wheelchairs can be efficient modes of transportation, chronic use and the lack of standing leads to several medical concerns not limited to osteoporosis, muscular atrophy, pressure ulcers, and bladder dysfunction [4–6]. The initial goal of developing exoskeletons for people with SCI was to help combat these risks by allowing the individuals to transfer out of the wheelchair, stand up, and walk again. Commercial devices from ReWalk [7], Ekso Bionics [8], and Indego [9] have made great advances on this front and represent the only devices to be approved by the U.S. Food and Drug Administration. However, use is limited to rehabilitation clinics or at home under the supervision of a trained spotter. As the exoskeleton research community continues to grow, especially since the introduction of the Cybathlon in 2016, the focus has shifted towards building devices that can be taken home and used safely, without supervision, throughout activities of daily living (ADLs) and beyond.

**Fig. 1:**
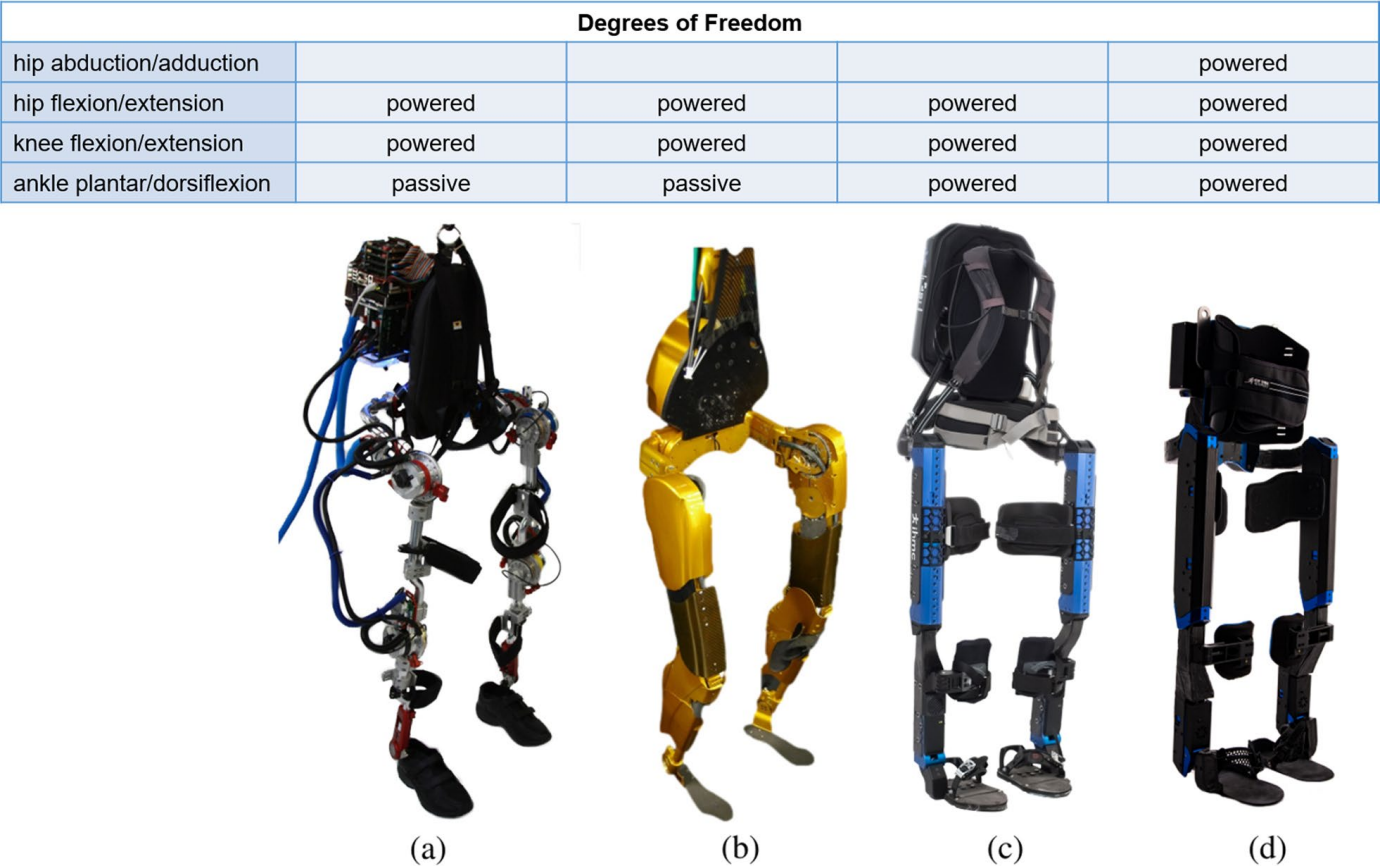
IHMC exoskeletons: **a** Mina v1, **b** X1, **c** Mina v2, used in 2016 Cybathlon, **d** Quix, used in 2020 Cybathlon

The devices from ReWalk, Ekso Bionics, and Indego certainly serve as inspiration to many of the research platforms that have followed. All three exoskeletons provide powered assistance through motors aligned with each hip and knee that allow users to sit, stand, and produce a variety of flat-ground walking gaits while balancing with forearm crutches. ReWalk and Indego users control when walking starts and stops by tilting their trunk forward or backward. The EksoGT from Ekso Bionics is controlled by the spotter and includes software that tracks the progression of a personalized rehabilitation program. The ReWalk is also capable of traversing stairs, though this functionality is not approved for use in the U.S.

The 2016 Cybathlon was an excellent display of the advancements that had been made by the top research labs and companies since the advent of these commercial devices. While ReWalk provided the performance benchmark at the time, both through pre-race demonstrations and as the winner of the competition, other noteworthy devices included TWIICE from EPFL, team Varileg, team SG Mechatronics (now Angel Robotics) with the WalkOn Suit, and the Institute for Human and Machine Cognition (IHMC) with Mina v2. This competition featured many new challenges faced by exoskeletons, including walking over stepping stones, up and down stairs, through a slalom course, and up ramps not compliant with ADA (Americans with Disabilities Act) specifications. This required many advances with respect to both usability and gait design, with the top performers being ReWalk, IHMC, and SG Mechatronics. However, no teams were able to complete all of the tasks, with only IHMC and ReWalk completing the stairs. Notable advances in this challenge included the incorporation of a powered ankle plantar/dorsiflexion joint by IHMC.

IHMC has been focused on the design and development of exoskeletons for individuals with SCI since 2010, with devices including Mina v1 [10], X1 [11], Mina v2 [12], and Quix, all shown in Figure 1. The original focus of IHMC’s exoskeleton development was an exploration of wearable robotics. However, focus quickly shifted to assistive devices for individuals with SCI. Mark Daniel has piloted all of these devices and been an integral part of the design iteration process. Daniel, age 31 at the time of the 2020 Cybathlon, suffered a complete T10-level SCI (ASIA Grade A) at the age of 18.

X1 was the first effort by IHMC to address the ease of use problem, resulting in a device that was easy to don and doff. The development of Mina v2 then led to a device that was easier to pilot and control, emphasizing a parameterized gait that could be tuned and developed online with user feedback and a powered ankle to reduce user effort. These design tenants were further enhanced in Quix, which utilized 3D body scans for a customized, user-centric design, added a powered hip ab/adduction joint to further decrease user effort, and streamlined the user interface. The gait was refined as well, in an attempt to emulate non-disabled walking while incorporating elements critical to robust exoskeleton walking like large ground clearance.

## Methods

**Fig. 2:**
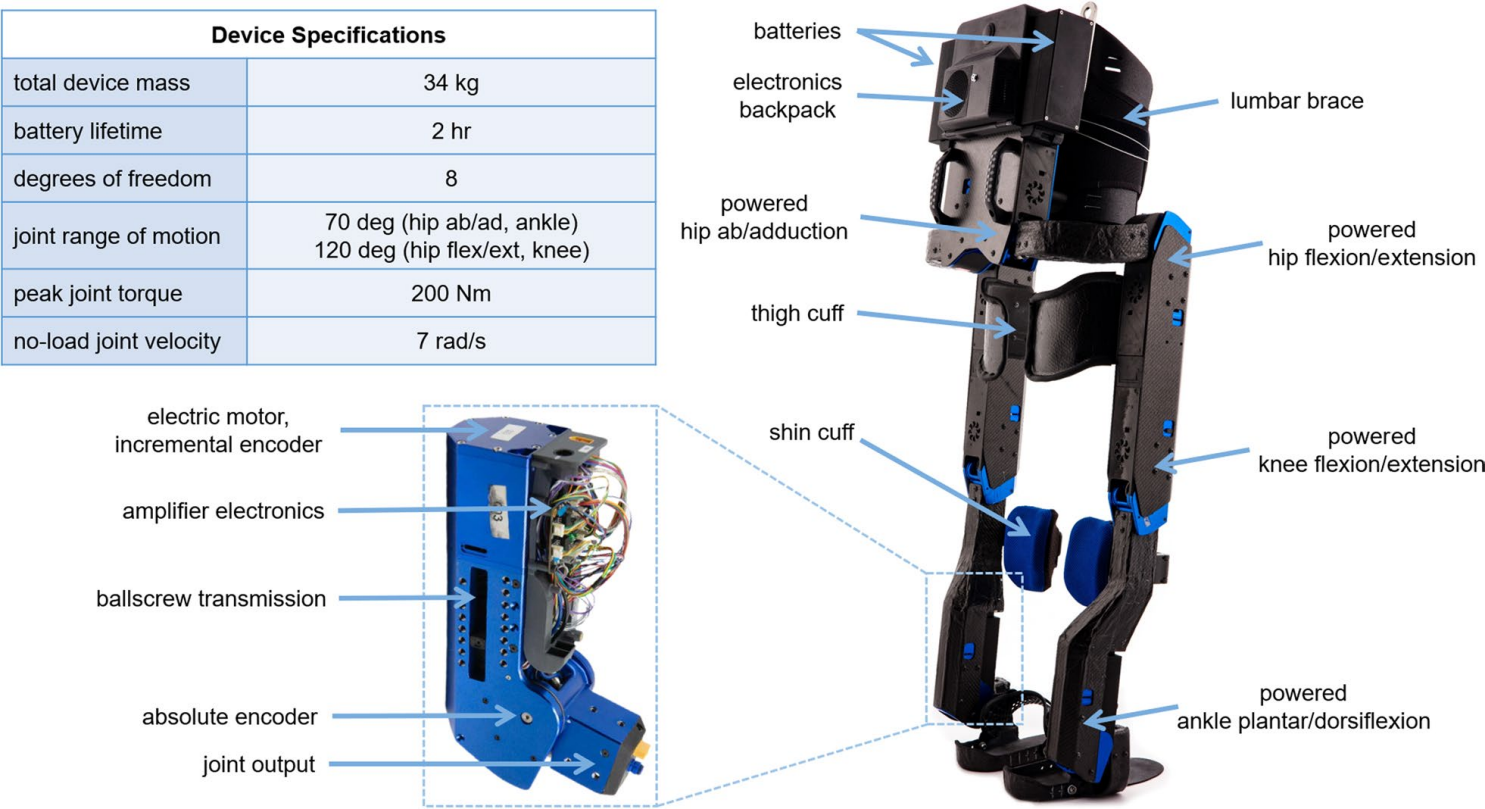
Key components of Quix exoskeleton

Quix is the latest exoskeleton from IHMC (Fig. 2), developed for competition in the 2020 Cybathlon and the Toyota Mobility Foundation’s Mobility Unlimited Challenge. The design builds upon the success from our previous devices and includes several key improvements, both in terms of device performance and user satisfaction. We strove to push the physical limits of the device while prioritizing user safety and comfort.

### Mechanical design

The structure of Quix is comprised mainly of the eight actuators and the carbon fiber sleeves that cover and connect them. Each leg includes four links and four actuators. The hip ab/adduction actuator positioned posterior to the pilot drives a carbon fiber tube that wraps around the waist and connects to the thigh link. This link includes a single carbon fiber sleeve that houses both actuators for hip and knee flexion/extension. The thigh then connects to the carbon fiber shank that houses the actuator for ankle plantar/dorsiflexion. Finally, the ankle drives the rotation of a carbon fiber foot plate that includes a rubber sole and straps, similar to those seen on snowboard bindings or roller-blades, that lock the pilot’s shoe in place. All carbon fiber pieces were fabricated in-house by covering 3D printed molds in pre-impregnated carbon fabric sheets that were then vacuum-sealed and cured in an oven.

Each of the eight actuators features an Allied Motion MF0060044 brushless motor driving a ballscrew transmission along a linear slide carriage with an output linkage that enables the joint rotation. We designed two versions of this linear-linkage actuator that differ in their ballscrew length, and hence, their size, weight, and range of motion. This was done in an attempt to reduce the mass placed at joints that exhibit relatively smaller ranges of motion. The shorter version, weighing approximately 2.3 kg, allows for 70 degrees of rotation and is used for ankle plantar/dorsiflexion and hip ab/adduction, while the longer version, weighing approximately 2.5 kg, allows for 120 degrees of rotation and is used for hip and knee flexion/extension. The range of motion can be further clamped with 3D printed hard-stops. Both versions are capable of producing a peak torque of about 200 Nm and a no-load velocity of 7 rad/s.

The remainder of the exoskeleton structure includes a 3D printed backpack for housing the computer, batteries, and other electronics, as well as the cuffs and straps used to secure the pilot to the device. A 3D scan of our pilot’s body was used to model the size and curvature of the carbon fiber cuffs placed behind his thighs and across his shins to minimize the imposed shear forces on soft tissue during operation. A layer of foam padding allows for a comfortable fit, and straps with single-hand-release buckles (BOA, Colorado, USA) facilitate quicker donning and doffing. Finally, a lumbosacral orthosis and waist strap (Top Shelf Orthopedics, California, USA) secure the pilot’s torso to the backpack.

**Fig. 3:**
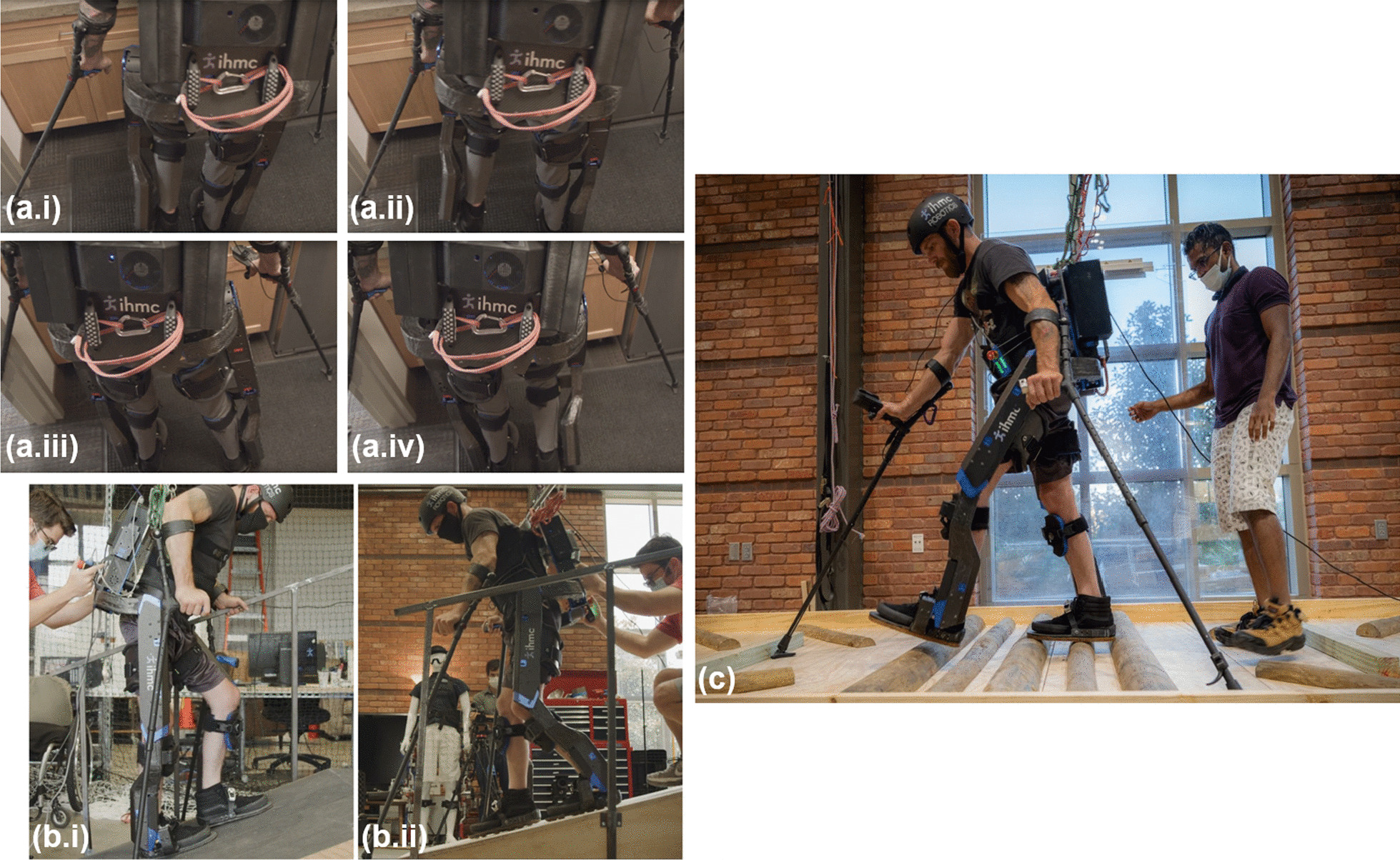
Quix pilot, Mark Daniel, performing various exoskeleton behaviors: ** a.i**–**a.iv** side-stepping sequence, **a.i** lifting left leg, **a.ii** swinging out left leg, **a.iii** lifting right leg, **a.iv** swinging in right leg; **b.i** walking up ramp, **b.ii** walking down ramp; **c** walking across rough terrain

Quix features a few notable design improvements since Mina v2 centered around ease of use and pilot comfort. The added degree of freedom (DoF) for hip rotation in the frontal plane allows the device to shift the weight of both itself and the pilot over the stance leg while walking, reducing the upper-body effort required to balance with the crutches. These new actuators also allow the exoskeleton to step directly side to side as shown in Fig. 3a. We additionally established a pipeline for personalizing the fit of the exoskeleton to our specific user. The 3D body scan enables custom fabrication of the leg cuffs, and a variety of mounting locations for both the cuffs and actuators allow us to more finely tune the fit with a resolution of 9 mm. Further, the torso brace replaces the backpack shoulder straps used on Mina v2, improving overall comfort and ease of donning and doffing. Finally, the reduced weight and more compact design of the backpack moves the overall center of mass (CoM) closer to the pilot’s, facilitating improved balance during standing and walking.

### Electrical design

Each actuator is outfitted with an electronics carrier board, featuring a Gold Twitter motor drive (Elmo Motion Control, Petah-Tikva, Israel) capable of executing position, velocity, or current control. The carrier board also breaks out connections for an array of on-board sensors and other mechanisms. These sensors include an RMB20 incremental magnetic encoder on the motor and an AksIM-2 absolute magnetic encoder on the output (RLS, Ljubljana, Slovenia), an LCB200 rod end load cell in line with the linkage output (Futek, California, USA), and four discrete temperature sensors placed within the stator. A noteworthy addition to the Quix actuators is a solenoid-operated mechanical brake. A circular aluminum tooth profile is attached to the solenoid output and faces a matching profile attached to the rotor. The solenoid stays energized during operation such that its teeth are held back from the rotor’s. It then de-energizes upon an emergency stop, software fault, or overheating motor, causing the teeth to collide and the rotor to abruptly slow to a halt. A mechanical override exists on each actuator to separate the teeth while the device is not powered so that joints can be backdriven for repositioning.

The remaining electronics are housed in the 3D printed backpack. Everything is powered by two 22,000 mAh batteries holstered on either side of the backpack. A power distribution board breaks out power to all peripherals and is equipped with protection for over-current and under-voltage. Real-time Java control threads run on an embedded computer (COM Express Type 6, ADLINK Technology Inc., New Taipei City, Taiwan) that communicates with each motor driver over EtherCAT. An EtherCAT junction box (Omron, Kyoto, Japan) splits off communication lines to each leg and a separate microcontroller that translates bidirectional signals between the main computer and a tethered pilot-safety box. The actuators along each leg are daisy-chained using snap-in IP67 connectors (Binder, Neckarsulm, Germany). In addition to an emergency-stop button that cuts current to the motors and engages the actuator brakes, the handheld pilot-safety box features status lights that indicate battery voltage level and potential device faults such as a motor overheating or a brake not releasing. During operation, the box is either secured to the pilot’s chest or in the hands of a spotter.

### Software design

Our software architecture is designed to run as a self-contained process in a real-time thread on the control computer. Abstraction barriers exist between the main control process and other sub-processes that enable behavior selection and parameter value updates from user input via data distribution service (DDS) messages. This allows the execution of footsteps and other movements to be commanded through the pilot interface or a remote computer.

Due to our use of Java, source code would be compiled and optimized at runtime using just-in-time compilation (JIT). Code blocks being optimized during JIT’s first pass would often be executed more slowly than post-optimization passes, causing missed deadlines on setpoint updates and resultant motor faults. To resolve this, we implemented a start-up routine that executed, and thus optimized, every branch of our code base without sending any setpoints to the hardware. As a further precaution, we would then execute all behaviors on the exoskeleton in the air before allowing pilot operation.

The different exoskeleton behaviors including sitting, standing, flat-ground walking, side-stepping, and ascending/descending ramps & stairs are separated into dedicated state machines. All elements of these different states, particularly the walking gaits, are designed specifically to enable online tuning of the exoskeleton trajectories through user feedback. The trajectories are parameterized using both setpoints that are specified via the external DDS commands and tuning parameters which can be updated online. These variables are synchronized online at 1 kHz with remote websocket clients. This allows the variables to be remotely viewed, modified, and logged, all in real-time and during operation. Variables for robot state are also included in this remote data synchronization process, enabling a reconstruction of the entire exoskeleton state during logging and remote visualizing.

### Trajectory design

All behaviors and respective trajectories utilize position control at all eight joints. The swing trajectory on Quix is designed in a similar fashion to that used by Mina v2 [12]. This method prescribes the swing-leg trajectory using four Cartesian position and velocity waypoints, including the start and end points along with two intermediate waypoints. For Quix, however, we designed these waypoints to be relative to the pelvis rather than in the world frame. The swing trajectory is parameterized by step length, initial foot position, waypoint fraction, waypoint height, and swing duration. We additionally specify a desired foot touchdown angle so that the swing foot is never flat when it makes contact with the ground. To compute the final swing trajectory, we minimize the intermediate waypoint velocities by varying the segment durations using an online gradient descent optimization.

As we noted in our previous work [12], the inclusion of powered ankle plantar flexion is critical to reducing the effort needed by the exoskeleton pilot, injecting energy into the system to propel the body forward [13]. For Quix, we also designed the transfer phase to “roll” onto the leading foot, with the foot settling to a flat position from its pitched angle on touchdown. In natural human walking, the energy injected by the ankle plantar flexion motion during the final portion of the toe-off phase acts to accelerate the swing leg forward [13]. We sought to replicate this behavior on Quix and introduced a novel “collapsing” action at the end of the loading phase, just prior to toe-off, of the trailing foot. This allows the toe-off motion to drive the trailing leg forward while collapsing, initiating the swing motion while still in the transfer phase. This puts the leg in a more optimal configuration at the start of swing, decreasing the required acceleration of the swing joints and induced torque about the CoM during the swinging motion.

We also sought to improve the walking phase by allowing toe-off to begin during the opposite leg’s swing phase. This toe-off action is present in natural human walking and has been linked to minimizing the energy loss during impacts on contact [14]. Beyond accelerating the CoM, moving more of this transfer duration into the swing phase minimizes the required time in transfer for the exoskeleton gait. Limiting the time spent in double support is critical to maximizing the overall walking speed. For reference, double support accounts for about 30% of the natural human gait cycle, which translates to a duration of less than 0.2 s at preferred walking speeds in adults not living with a disability [15–18].

To further decrease the required crutch forces for using Quix, we incorporated motion in the frontal plane using the powered hip ab/adduction joint. This is critical, as it laterally shifts the user’s weight over the stance foot during swing, which in turn reduces the required crutch force necessary to prevent the user from falling sideways. To do this, we modeled the lateral pelvis action using a sinusoidal motion, which is very similar to the hyperbolic motion of the CoM seen in non-disabled walking [19] and implemented on walking robots [20].

The stair and ramp trajectories used on Quix built directly off of the gaits designed for Mina v2 in the 2016 Cybathlon [12]. The most prominent change is again the hip ab/adduction motion to assist with shifting weight over the support foot when ascending/descending the stairs and ramps. An additional minor improvement included an improved ability to bias the user’s weight distribution forward and backward between steps on the ramps (Fig. 3b).

To ensure the resulting gaits are safe and reliable, we developed a rigid testing and evaluation procedure. First, the gait would be examined in simulation, both quantitatively and qualitatively to verify joint setpoint values and address any discontinuities. Then, the setpoints would be analyzed by running the exoskeleton in the air, further verifying that no excessively jerky motions would result. During this process, ranges of tuning variables would be assessed, confirming that the resulting motions were safe. From there, the exoskeleton would be worn and operated by an individual not living with a disability, with all changes being evaluated for performance and comfort. This also allowed initial values for the tuning variables to be determined. Only at this point was the exoskeleton determined to be safe for use by our pilot. During pilot evaluation, tuning variables were methodically modified, with specific feedback requested from the pilot on every change.

### Pilot interface

The control interface is housed in one of the forearm crutch handles. A 64x48 pixel OLED module (Microview, Sparkfun) displays textual messages representing permissible exoskeleton behaviors for the pilot to choose. The current list of displayed behavior options is based on the state of the exoskeleton, e.g., the option to walk is not available when the exoskeleton is in a seated position. Three push-buttons enable the pilot to scroll through options, confirm a behavior, and execute that behavior. Confirming a behavior relays the pilot’s intent to change state to the main computer and displays a unique submenu of options. For example, the submenu for standing guides the pilot through the three discrete motions programmed for completing the sit-to-stand movement. In the case of flat-ground walking, the submenu includes additional options for modifying step length and establishing intent to either manually trigger each individual step or to initiate a sequence of continuous steps that can then be manually triggered to stop. After all behavior-specific settings are confirmed, the execute button is the only one that can trigger movement of the exoskeleton. Using a dedicated button to initiate movement, as opposed to detecting user intent through other sensing modalities like an inertial measurement unit, for example, eliminates any uncertainty in the timing of the next initiated action. Both power and bidirectional serial data are tethered to the backpack via a single USB Type-A cable. A state machine running on an Arduino ATmega328P translates selected behaviors and parameter values to the main computer and updates the OLED display based on acknowledgement signals encoding exoskeleton state changes.

## Results

Most tasks from the 2016 Cybathlon were carried over to 2020 with a few notable changes. A manipulation task was added that tested the pilot’s ability to maintain balance while standing at a table and stacking cups. The stepping stones were replaced with a rough terrain obstacle featuring an array of unavoidable wooden logs. Finally, the tilted path was modified to include a single side-sloping ramp instead of two that sloped in opposite directions. We constructed the course in our lab space and dedicated anywhere from several days to a few weeks to practicing the movements involved with each obstacle. Our pilot began training approximately two months prior to the competition with most time being spent on tuning the ramp trajectories. The entire course was completed start to finish just five times prior to the Cybathlon.

### Sit & stand

Our state machine for sitting involves two discrete programmed motions that combine to create one fluid motion, such that pilot input is not required to transition between the two. The two motions and transition conditions are based on time durations to achieve sagittal-plane changes in hip, knee, and ankle angle. The hip ab/adduction actuators hold their zero positions during this motion. The first discrete motion aims to lower the CoM while keeping it above the base of support. The pilot places the crutches in front of the chair to protect from falling forward. Once the pilot is squatting just above the chair, the final motion rocks the device back into a seated position and flattens the foot plates on the ground.

The standing motion is split into three discrete motions, all requiring user input to transition. The goal here is to reduce the crutch force required from the pilot to shift the CoM over his feet before moving vertically. The first motion tucks the feet close to the front edge of the chair by flexing the knees and dorsiflexing the ankles to keep the foot plates flat on the ground. The second motion, along with force from the pilot’s crutches applied on either side of the chair, lifts the pilot and device out of the chair into a squatting position similar to that of the intermediate position during sitting. Without first tucking the feet under the knees, we found this lifting motion requires too much effort from both the device and pilot. As soon as the pilot feels balanced in the squatting position, he queues the transition to the final standing position.

In the interest of saving time during the competition, our pilot chose not to take a step forward towards the table to complete the manipulation task. He instead leaned the device forward while in a standing position, held both crutches with a single hand to balance, and stacked the cups. This made balancing quite challenging and we failed our first attempt at stacking the cups due to difficulty reaching.

**Fig. 4:**
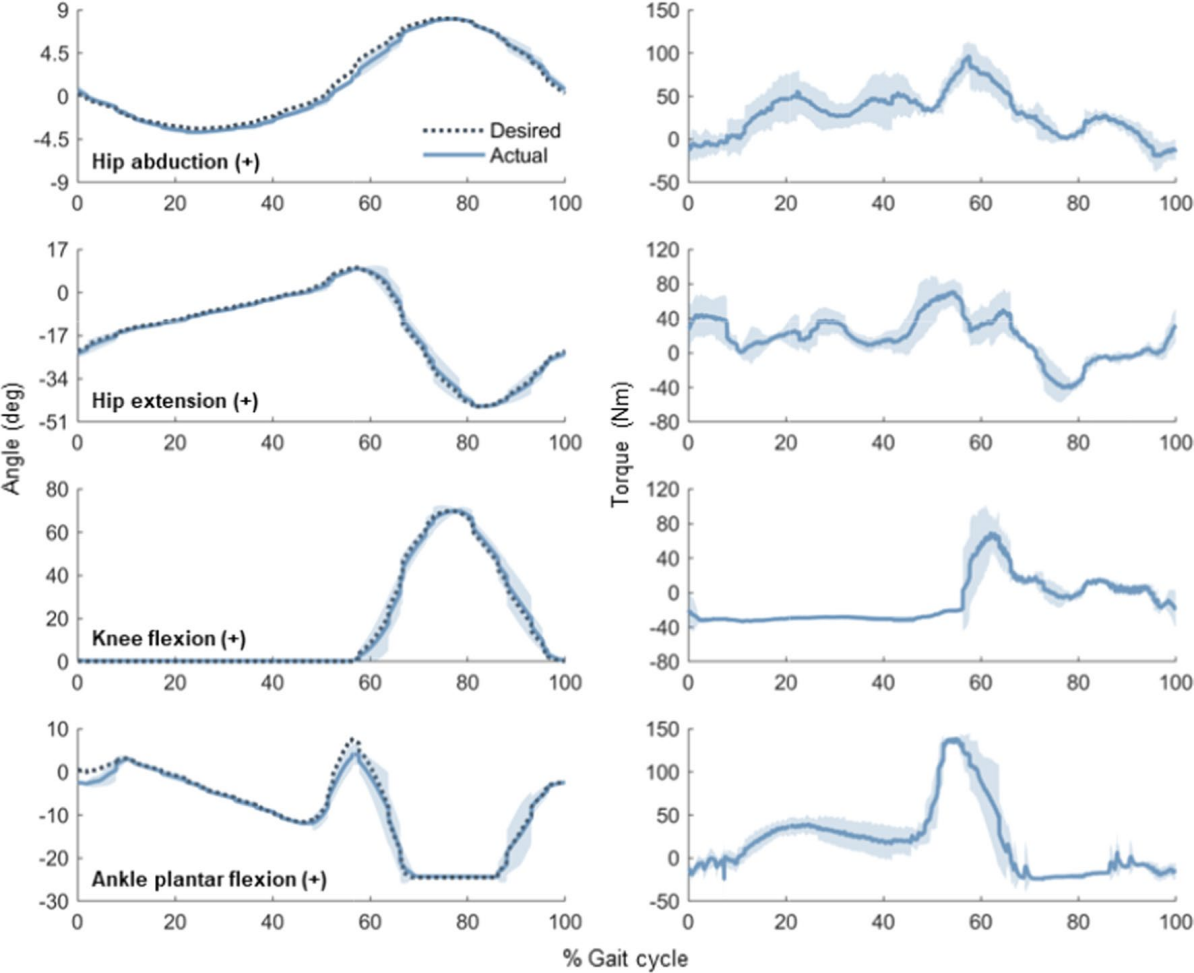
Joint angle trajectories and corresponding joint torques averaged over 5 continuous steps of 0.4 m in length, beginning at heel-strike. Shaded regions represent standard deviations of the actual, measured values

### Slalom, Rough terrain, Tilted path

Step-length options of 0.2 m, 0.4 m, and 0.59 m were initially included in the pilot interface for flat-ground training. While our pilot became comfortable with the longest steps during straight, flat-ground walking, especially after we modified the gait to reduce the time spent in double support, we decided that the extra length was not viable throughout all flat-ground sections of the Cybathlon. When maneuvering around tables during the slalom task, for example, the long steps caused the pilot to lose balance as he attempted to pivot the device on the stance foot during swing. We chose to use the medium-length steps throughout the entire slalom task to reduce the risk of instability and to eliminate time spent switching between lengths on the pilot interface. A plot of joint angle trajectories averaged over five continuous medium-length steps is shown in Fig. 4. The shorter step-length option was initially included as a means to make small adjustments in position before walking up the stairs, for example, but the pilot soon realized it was much quicker to utilize the crutches to lift the device off the ground just enough to shuffle forward.

When planning our control approach for the rough terrain and tilted path, we proposed several elaborate modifications to the flat-ground gait. These included different strategies for varying ankle impedance based on sensed foot placements over the rough terrain to help thrust the CoM forward before stiffening the ankle through toe-off. We also considered introducing offsets to the hip ab/adduction sine-wave controllers during traversal of the tilted path such that the pilot’s torso remained close to vertical to reduce the required force from the crutch closest to the ground. Upon request from our pilot, he first attempted these tasks using our standard flat-ground gait. He completed both with considerable ease and because it posed no evident safety risk, we chose not to over-complicate our control approach. Due to the predictability of the walking motion, our pilot was able to confidently generate momentum with his upper-body to help propel the device over the wooden logs on the rough terrain and fight the force of gravity during the swing phase on the tilted path. Long steps were used on the rough terrain to help aim for more stable landing surfaces, just based on the layout of the obstacles (Fig. 3c), but more time was taken between steps to maintain balance. Medium steps were used on the tilted path to further reduce the crutch force necessary to balance during swing.

Across these three tasks, we were slower than the winning team from Angel Robotics by a total of 1 min 52 s. Since we were only slower by 2 s on the rough terrain, the difference comes down to our continuous walking speeds. Quix can achieve a walking speed of about 0.25 m/s, while Angel Robotics reports a speed of 0.89 m/s [21].

### Stairs, Ramp & door

As previously noted, our trajectories for ascending and descending the stairs and ramps built directly off what was used on Mina v2 [12] with a few minor adjustments. As the stairs pose the most risk to the pilot, we were very careful to ensure each foot hold covered adequate surface area before initiating the next step. Further, we chose not to descend the stairs facing forward mainly because we were unable to prescribe a swing speed we deemed safe enough for pilot testing. A faster swing trajectory could result in a large impact force at touchdown if the device was not optimally positioned on the previous stair step. A slower swing trajectory would require the pilot to fight gravity while controlling the placement of the swinging foot for a longer period of time, which is physically demanding. This slower motion would also allow more time for the device to dangerously rotate forward about the stance foot. Our pilot instead executed a pirouette on the intermediate platform to prepare for a backward descent. The stair ascent trajectories were then played in reverse. This extra precaution contributed to us completing the stairs one minute slower than the fastest team. We did, however, reduce our time by 48 seconds compared to the same stairs task in the 2016 Cybathlon with Mina v2.

Aside from refining our flat-ground gait, most preparation was spent tuning the ramp trajectories. We focused specifically on defining double-support configurations that facilitated movement of the device in the desired direction. We initially struggled with our toe-off trajectory during ramp ascent propelling the device back down the ramp. This is a prime example of how our testing pipeline including a non-disabled user for preliminary trajectory evaluation saved valuable time and prevented our pilot from executing potentially risky movements. While the joint configurations prior to each step were essential to a successful ascent of the 20 degree ramp, we have seen that less attention to detail is necessary to descend the 15 degree ramp. Although our pilot successfully executed the ramp descent using the flat-ground gait in practice, we took extra precaution in competition and used trajectories designed specifically for the task. The winning team from Angel Robotics employed their flat-ground gait during the descent, leading to an overall task completion time that was 20 seconds faster than the next-best team. We again improved upon our time on this same task from the 2016 Cybathlon, traversing the ramps in 11 fewer seconds.

## Discussion

**Fig. 5:**
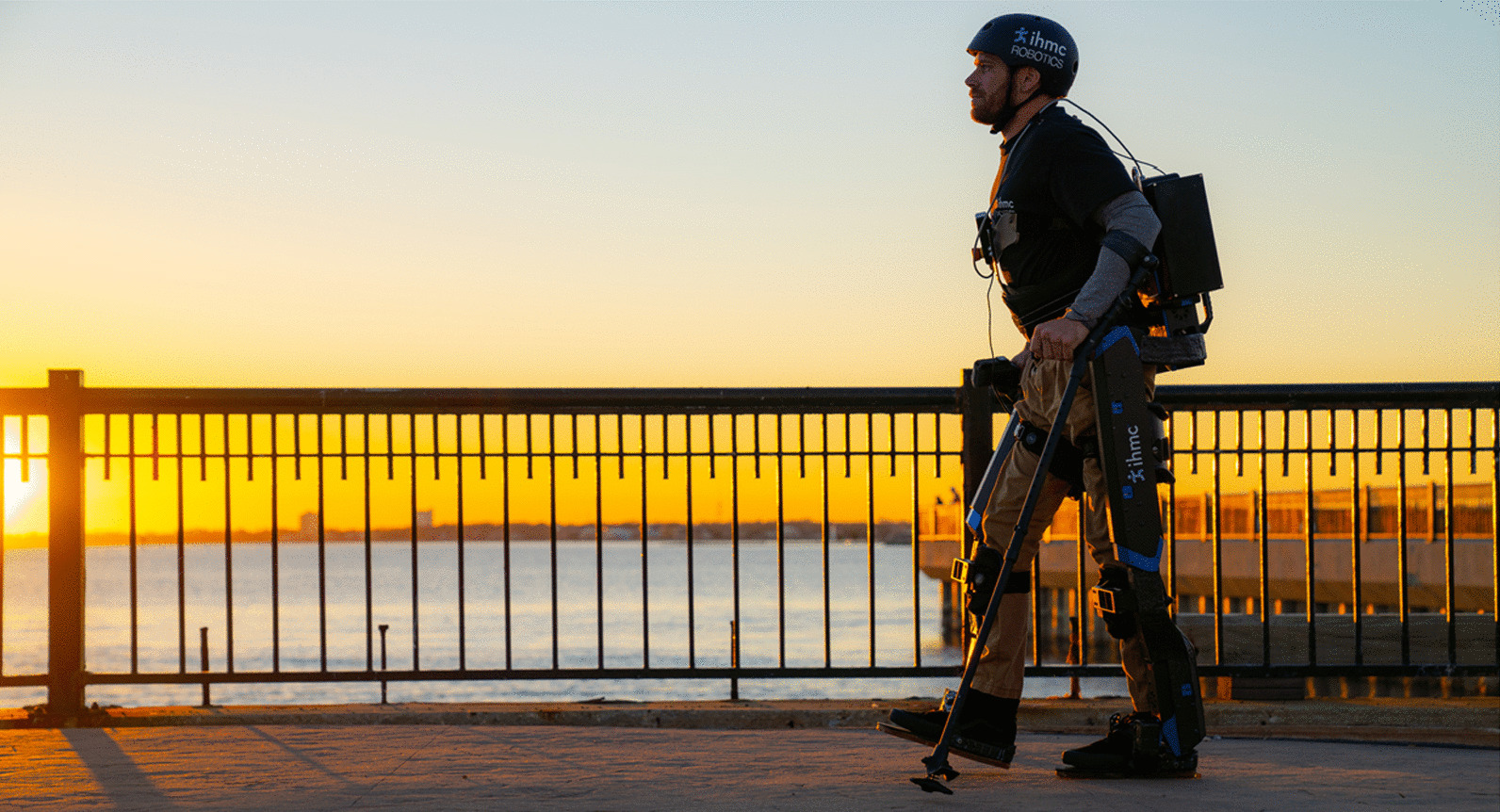
Mark Daniel walking with Quix along the Pensacola Bay after the 2020 Cybathlon

Team IHMC finished in fourth place at the 2020 Cybathlon. Our pilot, Mark Daniel, celebrated with a stroll along the Pensacola Bay at sunset, shown in Fig. 5. Compared to our device used in 2016, huge strides were made in reducing perceived user effort and improving overall comfort and ease-of-use. A noteworthy improvement was the streamlined pilot interface. With the intention of minimizing time spent operating the embedded crutch interface, we developed a competition-specific menu that attempted to provide options based on the pilot’s progression through the course. For example, the option to climb stairs was no longer available after completing the stairs task just to reduce any unnecessary button clicks. While there may not be a clear extension of this approach to real-world applications, additional sensing modalities could contribute to a more refined interface design. The integration of cameras, for example, could detect that the device is approaching stairs and give priority to those respective menu options, or detect that the user is standing close to a counter and will likely need to step backward or to the side.

**Fig. 6:**
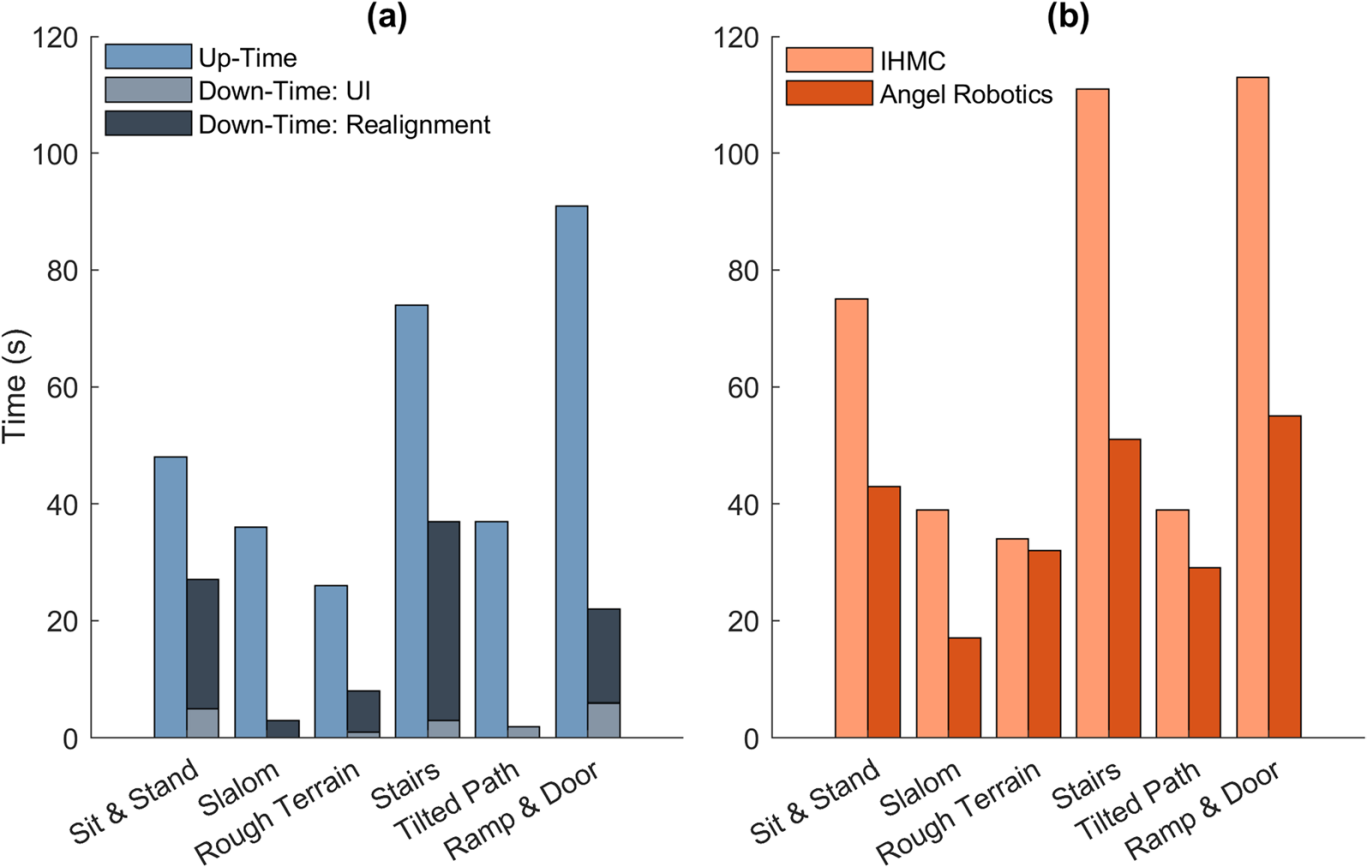
**a** Comparison of Quix up-time (progression through course) versus down-time (user-interface navigation or device realignment) during each task; **b** Comparison of total task time between IHMC and winning team, Angel Robotics

As shown in Fig. 6a, most of our down-time during the competition, essentially time spent not progressing through the course, is attributed to device realignment versus user-interface navigation. During the sit & stand and stairs tasks, realignment time accounted for roughly half of the overall task-completion time. These realignments involved our pilot using the crutches to manipulate the device in an attempt to establish a safe and balanced position before taking an additional step or sitting down. With the overall weight of the device nearing 34 kg, this realignment time is physically taxing. Due to the lengthy start-up routine to allow for sufficient JIT optimization, as well as the high joint torques required from our actuators during stair and ramp ascent, we were forced to use batteries each weighing as much as our heavier actuator.

Figure 6b illustrates just how dominant Angel Robotics was across the majority of the course, with only the rough terrain completion times being fairly equal. As previously mentioned, Daniel piloted Quix through the entire course five times prior to the competition. With his first few practice runs taking close to eight minutes to complete, Daniel soon figured out how to push the device to its limits and post a time of 6 min, 15 s in his final practice run followed by 6 min, 51 s in the final run of the competition.

Future work at IHMC will involve more quantitative comparisons between Mina v2 and Quix to better understand the apparent advantages of additional degrees of freedom and the benefits of powered versus passive joints. Plans include crutches instrumented with load cells to measure forces required for the user to balance as well as respirometry data collection to measure metabolic cost. Parallel efforts will include the addition of safety features such as center-of-pressure tracking during gait to ensure a proper forward weight shift has occurred before allowing a subsequent step.

## Conclusions

The ultimate goal in the advancement of exoskeletons for individuals with SCI and other gait pathologies is a device that is approved for safe and convenient everyday mobility. Until these devices can be quickly donned and doffed and enable efficient and non-strenuous navigation around a home and workplace, the wheelchair will continue to be the preferred option. If this ideal device did exist, there would still be an understandable safety concern surrounding unsupervised use. In short, this is a lofty long-term objective that will require a collaborative effort from the research community featured at the Cybathlon and beyond. As we progress onward, we should continue to improve the accessibility of the commercial devices in rehabilitation settings to help mitigate the noted health risks associated with chronic wheelchair use.

The powered exoskeleton race at the 2020 Cybathlon presented some format modifications tailored towards simulating ADLs, most notably the manipulation task involving stacking cups. A future extension of this may involve multiple manipulation tasks arranged along a countertop such that the most efficient movement is side-stepping. Quix includes a side-stepping behavior that was utilized in the Toyota Mobility Foundation’s Mobility Unlimited Challenge during a task simulating various ADLs. Another imperative requirement necessary to enable widespread adoption is minimized user effort, specifically the upper-body effort required to balance while walking. The challenge of actively balancing an exoskeleton with a human pilot has been successfully addressed by the teams behind Wandercraft [22] and REX [23]. Although these two devices do not require crutches for the user to balance, they consequently require six powered DoFs per leg and produce walking speeds limited to 0.15 m/s and 0.05 m/s, respectively. When compared to the 0.89 m/s reported by Angel Robotics [21] and a preferred human walking speed of about 1.3 m/s [15, 16], there is a necessary trade-off realized between level of balance assistance and safe walking speed. Wandercraft competed in 2020, featuring the first pilot without crutches. Though not able to attempt the rough terrain and tilted path, they did post competitive times on the stairs and ramps. While removing both crutches may not be feasible, as a result of limited walking speed and overall user trust, reduction to a single cane may be a potential compromise. Our pilot, who is in excellent physical condition, reported a sizable decrease in effort while walking due to the added hip ab/adduction actuators, but was still exhausted after a single run through the competition course. Though it is understandably difficult to add user effort to the criteria used to generate a score in the competition, a comparison between crutch forces across the various devices and obstacles would be an intriguing study. Static balance assistance while standing and performing a bimanual task, for example, may be a more practical step towards encouraging development in the direction of minimizing user effort.

Competitions like the Cybathlon have the unique opportunity and responsibility to design their tasks in a way that helps guide the direction of technological advancements. Enormous improvement was seen in the powered exoskeleton race between the 2016 and 2020 competitions, and we look forward to tackling the new challenges presented in 2024.

## Data Availability

No human-subject data was collected in this project. Exoskeleton data generated is available from the corresponding author on reasonable request.

## Abbreviations

SCI: Spinal cord injury
ADLs: Activities of daily living
IHMC: Institute for human and machine cognition
DoF: Degree of freedom
CoM: Center of mass
DDS: Data distribution service
JIT: Just-in-time compilation
